# Recent advances in positron emission tomography for detecting early fibrosis after myocardial infarction

**DOI:** 10.3389/fcvm.2024.1479777

**Published:** 2024-10-25

**Authors:** Qiuyan Wu, Jialin Song, Wenyan Liu, Li Li, Sijin Li

**Affiliations:** ^1^ Department of Nuclear Medicine, First Hospital of Shanxi Medical University, Taiyuan, China; ^2^ Collaborative Innovation Center for Molecular Imaging of Precision Medicine, Shanxi Medical University, Taiyuan, China; ^3^ Academy of Medical Sciences, Shanxi Medical University, Taiyuan, China

**Keywords:** myocardial infarction, myocardial fibrosis, positron emission tomography, molecular probes, fibroblast activation protein, αvβ3, collagen, proline

## Abstract

Cardiac remodeling after myocardial infarction is one of the key factors affecting patient prognosis. Myocardial fibrosis is an important pathological link of adverse ventricular remodeling after myocardial infarction, and early fibrosis is reversible. Timely detection and intervention can effectively prevent its progression to irreversible ventricular remodeling. Although imaging modalities such as CMR and echocardiography can identify fibrosis, their sensitivity and specificity are limited, and they cannot detect early fibrosis or its activity level. Positron emission tomography (PET) allows non-invasive visualization of cellular and subcellular processes and can monitor and quantify molecules and proteins in the fibrotic pathway. It is valuable in assessing the extent of early myocardial fibrosis progression, selecting appropriate treatments, evaluating response to therapy, and determining the prognosis. In this article, we present a brief overview of mechanisms underlying myocardial fibrosis following myocardial infarction and several routine imaging techniques currently available for assessing fibrosis. Then, we focus on the application of PET molecular imaging in detecting fibrosis after myocardial infarction.

## 1 Introduction

Adverse ventricular remodeling after myocardial infarction (MI) and its consequent left ventricular dysfunction and heart failure are the leading causes of death in patients with MI (1). Myocardial fibrosis is one of the core pathophysiological aspects of adverse ventricular remodeling after MI, which is characterized by excessive deposition of extracellular matrix (ECM) proteins leading to interstitial dilatation of the myocardium. This adverse ventricular remodeling is closely associated with cardiac diastolic and systolic dysfunction, arrhythmias, and a poor prognosis (2, 3). Although significant progress in the medical literature on the mechanism of post-MI fibrosis has revealed many potential therapeutic targets, clinical practice tends to focus more on improving myocardial function deterioration than on the fibrotic pathological process (4). Currently, treatment following MI still primarily relies on traditional drugs, such as angiotensin-converting enzyme inhibitors (ACEI), aldosterone antagonists, and β-blockers. Although these drugs can alleviate symptoms to some extent, they fail to provide personalized treatment based on individual patient differences, thus affecting their overall therapeutic effects (4). Anti-fibrotic therapy after MI remains a pressing therapeutic issue. Recent studies have shown that new anti-fibrotic treatments, such as pirfenidone pharmacotherapy and CAT-T cell therapy, have shown great potential (5, 6). Therefore, accurate assessment and comprehensive understanding of myocardial fibrosis are essential. Commonly used clinical imaging tools such as cardiac magnetic resonance (CMR) and echocardiography can aid in diagnosing fibrosis but have low sensitivity for detecting early fibrosis and its activity level. When mature fibrosis is detected, no appropriate treatment is frequently available (7, 8). Positron emission tomography (PET) is a non-invasive imaging technique that enables the more direct and specific detection of post-MI fibrosis activity by utilizing molecular probes that target fibrotic tissue. This technique can serve as a surrogate marker to identify high-risk individuals and determine the appropriate time to initiate treatment. In this review, we describe the pathophysiology of post-MI fibrosis, provide a brief overview of other non-invasive imaging techniques and focus on PET imaging assessment of MI fibrotic activity and its current research advances.

## 2 Post-MI fibrosis

**Figure 1:**
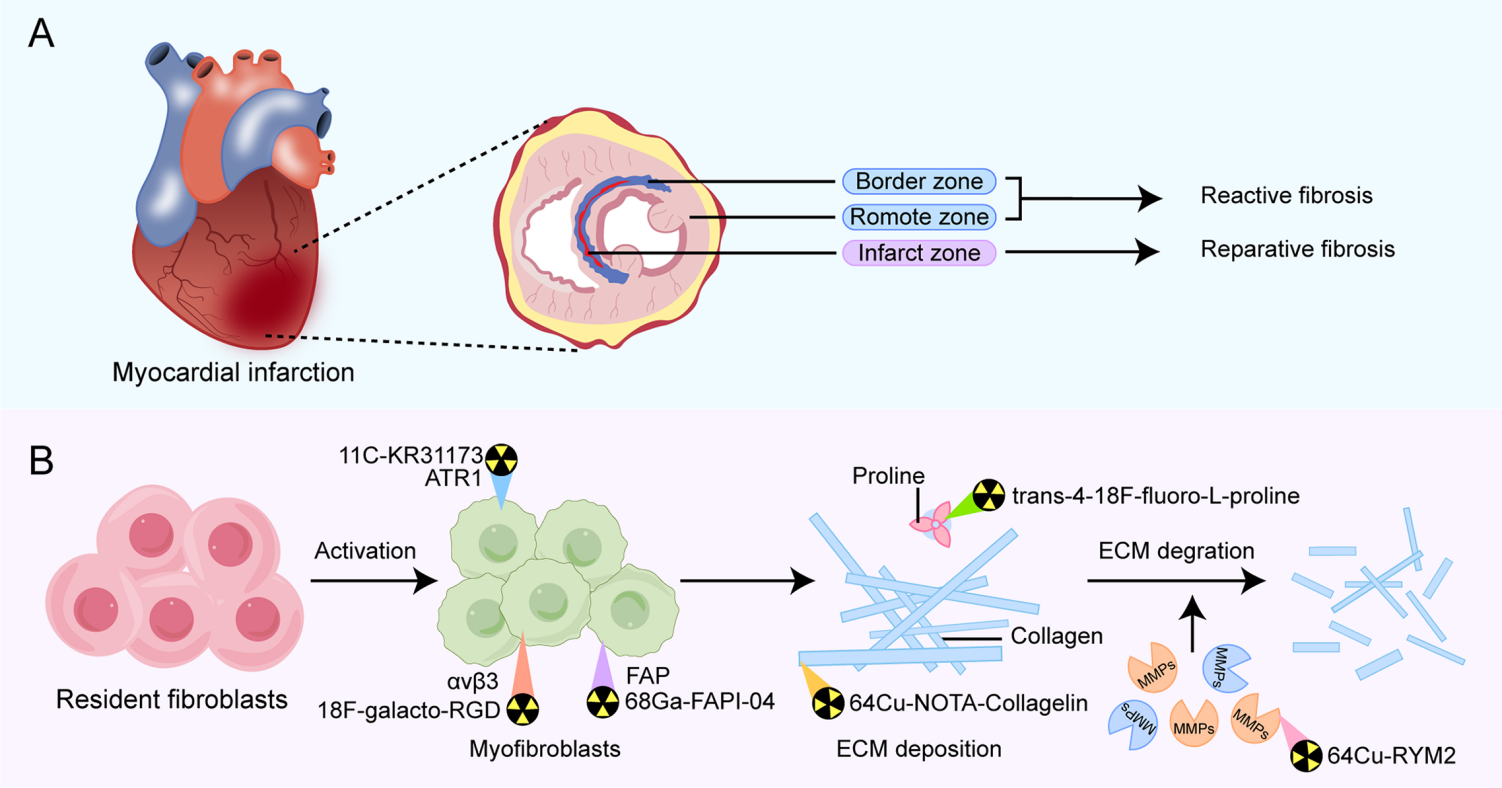
Overview of types of fibrosis occurring in different regions of post-MI fibrosis **(A)** and pathophysiological pathways in fibrotic activity for emerging PET tracers that have diagnostic and prognostic function **(B)**. Examples of the primary PET radiotracers for each target are shown above. MMPs, matrix metalloproteinases; FAP, fibroblast activation protein; ATR1, angiotensin receptor type Ⅰ.

Myocardial fibrosis is a critical pathological process involved in the development and progression of various cardiac diseases. The pathological mechanism for this is that healthy myocardial fibroblasts differentiate into pathological myofibroblasts under mechanical strain or the action of inflammatory stimuli and secrete excessive extracellular matrix (ECM), leading to remodeling of myocardial tissue structure (9, 10). Based on the characteristics and location of ECM protein deposition, myocardial fibrosis following MI can be mainly categorized into two main types: reparative fibrosis and reactive fibrosis (Figure 1A).

Reparative fibrosis predominantly occurs within the infarct zone, where it manifests as the replacement of necrotic cardiomyocytes with fibrotic scar formation, helping protect the heart from rupture. Reactive fibrosis is characterized by diffuse and disproportionate collagen deposition around the myocardial interstitium and blood vessels in the border zone of infarction and the distal non-infarcted myocardium. This type of fibrosis is attributed to incomplete inflammation resolution or an overactive reparative response (11, 12). The exact mechanisms of reactive fibrosis have not been completely elucidated, but increased mechanical strain on the non-infarcted left ventricular wall is one contributing factor. Additionally, persistently activated myofibroblasts within the infarct scar release pro-fibrotic factors, which may traverse remote myocardial regions, promoting local fibroblast activation and proliferation, thereby increasing collagen deposition in the interstitium space (13).

Unstable reparative fibrosis can result in the formation of a low tensile strength scar, which promotes ventricular dilatation and contributes to the development of systolic heart failure. On the other hand, excessive fibrotic activation may exacerbate reactive fibrosis in the infarct border zone, which in turn increases the risk of diastolic dysfunction (14). The optimal therapeutic approach for MI patients is to maintain moderate early fibrosis to preserve the structural integrity of the heart while suppressing late excessive fibrosis to prevent adverse ventricular remodeling. To date, only a few antifibrotic therapies can slow the progression of fibrosis, and they are generally unable to reverse established fibrosis (7). Therefore, selecting an appropriate non-invasive imaging technique to detect and visualize early fibrosis is crucial for guiding timely therapeutic intervention.

## 3 Non-invasive imaging techniques

Myocardial fibrosis can be comprehensively evaluated in a non-invasive manner using modern imaging techniques, including cardiac magnetic resonance (CMR), echocardiography, and nuclear imaging. Cardiac computed tomography can also be used to evaluate fibrosis, but due to its limited clinical application, it will not be discussed further. The imaging techniques available for clinical practice are summarized in Table 1.

**Table 1:** Non-invasive imaging techniques for assessment of myocardial fibrosis.

Imaging techniques	Advantages	Disadvantages
CMR (LGE, T1 mapping, ECV)	• Multi-parameter assessment of cardiac structure and function • Gold standard • LGE for focal fibrosis • T1 mapping and ECV for diffuse fibrosis	• Presence of contraindication • Lack of robust standardization and reference range • Not specific for fibrosis • Identify fibrosis at a relatively late stage
Echocardiography (TDI, STI)	• Low cost • High accessibility • Multi-parameter assessment of cardiac structure and function	• Operator-dependent • Not specific for fibrosis • Identify fibrosis at a relatively late stage • Inability to identify fibrosis type and quantify fibrosis
SPECT (Targeted probes for collagen, αvβ3, and ATRI…)	• Detect fibrosis at an early stage • Molecular specificity • Relative quantification	• Radiation exposure • Low spatial resolution • Lack *in vivo* human experience
PET (Targeted probes for FAP, αvβ3, and collagen…)	• High sensitivity • Detect fibrosis at an early stage • Molecular specificity • Absolute quantification	• Radiation exposure • High cost • Limited availability of tracers • Clinical application is limited and requires further exploration of its exact value

LGE, delayed gadolinium enhancement imaging; ECV, extracellular volume fraction; TDI, tissue doppler echocardiographic imaging; STE, speckle tracking echocardiography; FAP, fibroblast activation protein; ATRⅠ, angiotensin receptor type 1.

### 3.1 Cardiac magnetic resonance (CMR)

CMR is considered the gold standard for diagnostic imaging of myocardial fibrosis, as it allows non-invasive qualitative and quantitative assessment of focal and diffuse myocardial fibrosis (15). Delayed gadolinium enhancement imaging (LGE), T1 mapping, and extracellular volume fraction (ECV) are commonly used techniques in CMR for assessing myocardial fibrosis.

LGE has been regarded as the clinical standard for evaluation of focal myocardial replacement fibrosis. Both the expansion of extracellular space and a decrease in clearance rates can result in the retention of gadolinium-based contrast agents (GBCAs) within fibrotic areas. Increased gadolinium uptake in myocardial infarction (MI) scar, compared to normal myocardium, causes focal areas with a higher signal intensity, which corresponds to macroscopic scar observed on histology in both the acute and chronic phases of infarction (16). However, when fibrosis is diffuse and evenly distributed, the difference in signal intensity between fibrotic and normal myocardium is slight, which makes it difficult for LGE to distinguish fibrotic areas from normal myocardium. Therefore, the application of LGE in assessing diffuse interstitial fibrosis is restricted (17). Moreover, LGE is influenced by various technical parameters and is not reliable for quantitative assessment of myocardial fibrosis (18). In general, LGE is mainly used for qualitative assessment of fibrosis regions by observing high signal areas to determine whether fibrosis exists.

T1 mapping and ECV are advanced CMR relaxometry techniques that allow quantitative measurement of myocardial interstitial fibrosis. T1 mapping can be performed before or after the administration of gadolinium-based contrast agents to quantify the exact T1 of tissue, which reflects changes in tissue characteristics. Native T1 describes the signal within the myocardial tissue, thereby representing a combined signal from all components present. Fibrosis can cause local or global changes in signal intensity, resulting in an increase in T1 value (19, 20). Native T1 is reproducible and, more importantly, does not need gadolinium contrast medium, which allows its use in MI patients with renal dysfunction. T1 mapping can also be used in combination with the administration of gadolinium, which increases proton relaxation and reduces T1 relaxation time. Post-contrast T1 values are lower in the presence of myocardial fibrosis, in contrast to the native T1 relaxation time (19, 20). Both native and post-contrast T1 mapping can identify an increase in ECM volume that is not detectable by LGE, thereby reflecting interstitial fibrosis. The calculation of ECV relies on the ratio of the contrast agent concentration in the tissue to that in blood (i.e., the partition coefficient). Fibrotic tissue generally has a higher ECV, indicating an expanded extracellular space capable of retaining more contrast agents (21). Coregistration by T1 mapping can also generate pixel-wise ECV maps to locate diffuse fibrosis (22, 23).

CMR is the leading technique for the diagnosis of myocardial fibrosis. Also, it provides a comprehensive assessment of cardiac structure and function, including chamber size, vessel diameters, blood flow, and myocardial relaxation times (24). Although it has been widely accepted as a well-established non-invasive imaging technique, there are also several limitations. First, the application of CMR is significantly restricted for patients with implanted medical devices and those who have claustrophobia. Second, CMR-obtained parameters such as LGE, T1, and ECV measure extracellular expansion rather than fibrosis itself. While extracellular expansion is usually caused by fibrosis, it can also result from other pathological processes, including oedema, infiltration, and protein deposition (25, 26). Therefore, CMR-derived parameters can serve as surrogate markers of fibrosis only after other potential causes of extracellular expansion have been excluded. Third, standardizations in data acquisition and postprocessing are lacking for T1 mapping, which may result in variations in results depending on the equipment, imaging parameters, and analysis methods used (27, 28).

### 3.2 Echocardiography

Echocardiography is a user-friendly, cost-effective, and portable imaging technique used more frequently in clinical settings to assess cardiac morphology, structure, and function. It lacks any relative contraindications. In MI patients, echocardiography can be used to assess parameters such as left ventricular ejection fraction (LVEF), regional wall motion abnormalities (RWMA), and chamber size and to identify complications, including ventricular aneurysms, cardiac rupture, and papillary muscle dysfunction (29).

Regional strain is a dimensionless measure of myocardial deformation that quantifies myocardial function by expressing the fractional or proportional change in myocardium relative to its original size during deformation. Strain rate is the speed at which myocardial deformation occurs. These parameters allow earlier detection of functional abnormalities during myocardial fibrosis. While both echocardiography and CMR can measure myocardial strain, echocardiography-derived strain is more widely used in clinical settings to detect, monitor, and guide interventions in various cardiac conditions (30, 31). Compared to conventional echocardiographic techniques, Tissue Doppler echocardiographic imaging (TDI) and speckle tracking echocardiography (STE) are more helpful in measuring myocardial strain during the early stages of myocardial fibrosis. These advanced echocardiographic imaging techniques can detect strain abnormalities before fibrosis causes significant myocardial functional decline, indirectly indicating the presence of fibrosis (32, 33). Strain imaging has proven beneficial in patients with MI. In particular, global longitudinal strain (GLS) has been shown to be associated with fibrosis, poor remodeling, and prognosis (31).

However, it is worth mentioning that echocardiography does not specifically detect fibrosis or accurately determine its underlying type. Therefore, it cannot be used independently to measure and monitor the extent and progression of myocardial fibrosis. In addition, the results of echocardiographic assessment are highly dependent on the operator's skill level and experience, resulting in poor reproducibility.

### 3.3 SPECT molecular imaging

While the imaging techniques mentioned above can provide information about the formed fibrosis and its alternative indicators, they cannot detect the early stages of fibrogenesis and give information on ongoing disease activity. As an emerging noninvasive tool, nuclear imaging with positron emission tomography (PET) or single-photon emission computed tomography (SPECT) can visualize fibrotic processes at cellular and subcellular levels. Therefore, targeting the molecules or proteins involved in pro-fibrotic activity can improve the accuracy of early post-MI fibrosis diagnosis (34, 35).

Several radiotracers developed for molecular imaging have been employed to directly or indirectly identify early post-MI fibrosis in multiple animal studies by SPECT (36). Muzard et al. (34) designed a new and original specific probe for collagen, ^99m^Tc-labeled collagelin. In a rat model of MI, *in vivo* SPECT imaging showed significant tracer uptake in regions where fibrosis was histologically confirmed but not in controls. SPECT imaging with ^99m^Tc-labeled CRIP can identify myocardial fibrosis activity indirectly by targeting αvβ3 integrins expressed on cardiac fibroblasts as well as other cell types, such as endothelial cells and macrophages (37–39). In a murine model of MI, ^99m^Tc-CRIP uptake peaked 2 weeks after myocardial injury and then gradually decreased (37). Tracer uptake also correlated negatively with echocardiographic left ventricular ejection fraction and strain parameters and was related to histological collagen fiber deposition (40). Angiotensin II type-1 receptor (AT1R) expression is linked to interstitial fibrosis. SPECT imaging with ^99m^Tc-losartan targeting AT1R has also been proven feasible in a murine model (41). Although numerous SPECT imaging studies have been conducted on post-MI fibrosis in animal models, there is still a lack of experience in human subjects. Consequently, it is challenging to conduct a comprehensive evaluation of the cardiac kinetics and safety of these molecular probes in human subjects.

### 3.4 PET molecular imaging

PET imaging has higher spatial resolution and sensitivity than SPECT imaging, allows for absolute quantification, and is often combined with CT or MRI to provide a more detailed fusion of functional and anatomical images (42). However, the associated radiation dose with PET should be carefully considered, as it varies depending on the specific radiotracer and protocol used. Similarly, SPECT imaging also involves radiation exposure. When PET or SPECT is combined with CT for fusion imaging, which is frequently done to provide accurate anatomical localization, the total radiation dose is the sum of both modalities (43). Despite the concern over radiation exposure, the superior specificity and sensitivity of PET imaging make it more effective for early lesion detection and diagnosis.

In recent years, with the development of molecular probes targeting fibrosis-specific or fibrosis-associated processes, PET imaging has become an active area of research in cardiovascular diseases (44). PET molecular imaging can non-invasively monitor and quantify early post-MI myocardial fibrosis, contributing to an in-depth understanding of the fibrosis progression rate or regression. The identification of key molecules and proteins involved, such as fibroblast activation proteins, integrin receptors, collagens, and their precursors, using targeted tracers (Figure 1B), may provide valuable information about the activity or mechanisms of post-MI fibrosis, enable early diagnosis of fibrosis, guide clinical practice, and facilitate early antifibrotic therapy as well as earlier assessment of therapeutic response. Preclinical and clinical studies in MI have been conducted on PET imaging for these targets, some of which have progressed (Table 2). Here, we will discuss current research advances in PET imaging for different targets in detecting myocardial fibrosis after MI.

**Table 2:** PET imaging to identify myocardial fibrosis after myocardial infarction.

Target	Probe	Application	Reference
FAP	^68^Ga-FAPI-04	Preclinical（rat MI model)/Clinical	(35, 45)
	^68^Ga-MHLL1	Preclinical (mouse MI model)	(46)
	^68^Ga-FAPI-46	Clinical	(47, 48)
	^18^F-AlF-NOTA-FAPI-04	Clinical	(49)
	^68^Ga-DOTA-FAPI-04	Clinical	(50)
αvβ3 integrin	^18^F-galacto-RGD	Preclinical (rat MI model)/Clinical	(51–54)
	^68^Ga-NODAGA-RGD	Preclinical (mouse MI model/rat MI model/pig MI model)/Clinical	(53, 55–57)
	^18^F-Fluciclatide	Clinical	(58)
	^68^Ga-DOTA-E[c(RGDyK)]2	Preclinical (rat MI model/pig MI model)/Clinical	(59–63)
	^18^F-AlF-NOTA-PRGD2	Preclinical (rat MI model)	(64)
	^68^Ga-TRAP(RGD)_3_	Preclinical (rat MI model)	(53)
Collagen	^64^Cu-NOTA-Collagelin	Preclinical (rat MI model)	(65)
	^64^Cu-NOTA-CRPA	Preclinical (rat MI model)	(65)
Proline	trans-4-18F-fluoro-L-proline	Preclinical（rat MI model)	(66)
Angiotensin II receptor Ⅰ	^11^C-KR31173	Preclinical (pig MI model)	(67)

#### 3.4.1 PET imaging of FAP

The healing process of post-MI cardiac wounds undergoes pro-inflammatory to anti-inflammatory to reparative responses, and cardiac (myo)fibroblasts play a pivotal role in the process (14). Fibroblast activation protein (FAP), a membrane-bound serine protease, is highly expressed in activated (myo)fibroblasts and is closely associated with myocardial fibrosis (68). Fibroblast activation protein inhibitor (FAPI) binds to FAP with high affinity and specificity, and PET molecular imaging using radio-labeled FAPI can visualize activated fibroblasts and identify early active fibrosis. In recent years, FAPI PET molecular imaging has been applied in MI (69). Table 3 lists relevant studies and key findings of PET molecular probes targeting FAP in post-MI fibrosis.

**Table 3:** FAPI PET imaging in post-myocardial infarction fibrosis.

Probe	Application	Key findings	Reference
^68^Ga-FAPI-04	Preclinical (AMI rats, *N* = 20)	• FAPI uptake in the MI territory peaked 6 days after coronary artery ligation, then gradually declined, returning to baseline level after 2 weeks. • Tracer uptake was higher in the infarct border zone than in the infarct zone and remote non-infarct zone.	(35)
^68^Ga-MHLL1	Preclinical (AMI mouse, *N* = 14)	• FAPI signal in the infarct and border zone persisted up to 21 days after AMI. • FAPI signal was observed in the remote non-infarction myocardium.	(46)
^ 68^Ga-FAPI-46	Clinical (STEMI patients, *N* = 5, NSTEMI patients, *N* = 5)	• Affected myocardium showed a partial to complete match between tracer uptake and confirmed culprit lesion by coronary angiography. • FAV strongly correlated with peak creatine kinase levels and negatively correlated with left ventricular function.	(47)
^ 68^Ga-FAPI-46	Clinical (STEMI patients, *N* = 35)	• FAPI uptake significantly exceeded the infarcted myocardium area. • FAP signal does not match regional CMR tissue characteristic. • FAP volume significant negatively correlated with LV ejection fraction obtained at later follow-up.	(48)
^ 18^F-AlF-NOTA-FAPI-04	Clinical (STEMI patients, *N* = 14)	• FAPI uptake exceeded the area of edema and infarcted myocardium. • In the area of MVO identified by LGE, FAPI imaging showed lower FAPI uptake compared with the surrounding myocardium.	(49)
^ 68^Ga-DOTA-FAPI-04	Clinical (STEMI patients, *N* = 26)	• Compared with baseline clinical characteristics, CMR imaging, and cardiac function parameters, FAPI uptake volume showed superior predictive ability in predicting late left ventricular remodeling 12 months after AMI. • FAPI uptake can still be detected 12 months post-MI.	(50)

AMI, acute myocardial infarction; STEMI, ST-segment elevation myocardial infarction; NSTEMI, Non-ST-segment elevation myocardial infarction; FAPI, fibroblast activation protein inhibitor; FAV, fibroblast activation volume; CMR, cardiac magnetic resonance; LV, left ventricular; MVO, microvascular obstruction; LGE, Delayed gadolinium enhancement imaging.

Varasteh et al. (35) first used ^68^Ga-FAPI-04 PET imaging to study the dynamics of fibrosis in acute myocardial infarction (AMI) rats at different time points and found that FAPI uptake in the MI territory peaked 6 days after coronary artery ligation then gradually declined, returning to baseline level after 2 weeks. Tracer uptake was higher in the infarct border zone than in the infarct and remote non-infarct zones. They did not observe increased uptake of ^68^Ga-FAPI-04 in the remote non-infarcted myocardium. Langer et al. (46), using ^68^Ga-MHLL1 (another FAP-targeted tracer) imaging of AMI mice, obtained similar results, and they observed FAPI uptake in the infarct zone and the border zone persisted up to 21 days after AMI. Interestingly, Unlike the former study, they observed a signal of ^68^Ga-MHLL1 in remote non-infarction myocardium, indicating the presence of reactive fibrosis. One possible reason for this is that the infarction in MI rats in the former study occurred over a short period and had not yet progressed to the stage of heart failure, resulting in reactive fibrosis that may be weak or not yet apparent. Another possibility is that ^68^Ga-FAPI-04 PET imaging is not sensitive enough to identify early minimal fibrosis (35, 45). These animal studies support the feasibility of FAPI PET in early post-MI reparative fibrosis imaging.

**Figure 2:**
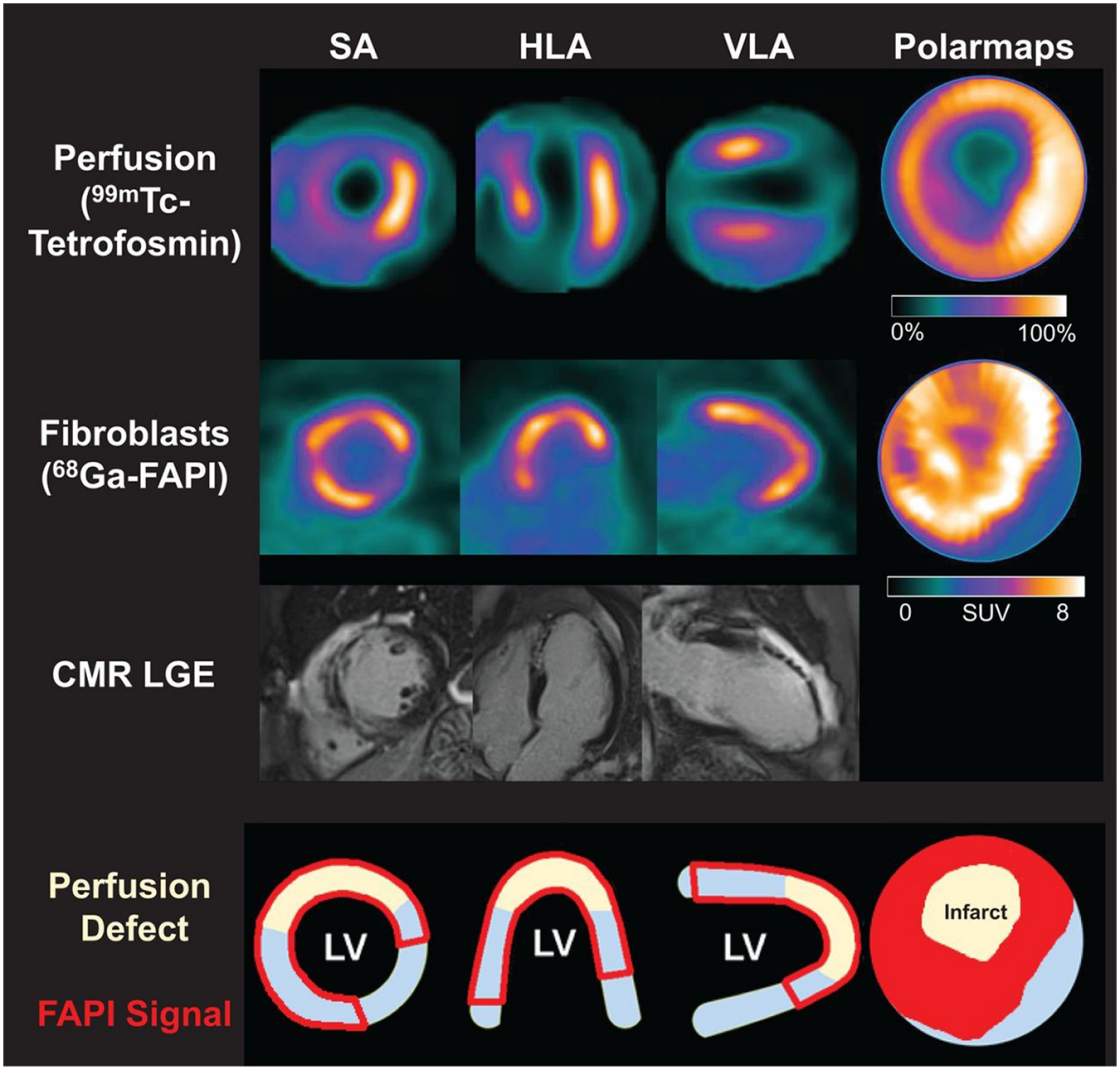
Myocardial perfusion imaging show a perfusion defect which indicating infarct zone. Area of fibroblast activation as indicated by ^68^Ga-FAPI-46 PET signal exceeds infarct area and LGE signal. HLA, horizontal long axis; SA, short axis; VLA, vertical long axis. Reprinted with permission from “Myocardial perfusion images using 99mTc-tetrofosmin at rest, 68Ga-FAPI PET, LGE from CMR, and schematic drawings of LV. Area of fibroblast activation as indicated by 68Ga-FAPI-46 PET signal exceeds infarct area and LGE signal, the most common type of myocardial FAP distribution. HLA = horizontal long axis; SA = short axis; VLA = vertical long axis” by Johanna Diekmann, Tobias Koenig, James T. Thackeray, Thorsten Derlin, Christoph Czerner, Jonas Neuser, Tobias L. Ross, Andreas Schäfer, Jochen Tillmanns, Johann Bauersachs and Frank M. Bengel, licensed under CC BY 4.0. This research was originally published in JNM. Cardiac Fibroblast Activation in Patients Early After Acute Myocardial Infarction: Integration with MR Tissue Characterization and Subsequent Functional Outcome. J Nucl Med. 2022;63:1415-1423 © SNMMI.

Kessler et al. (47) conducted ^68^Ga-FAPI-46 PET imaging in 10 AMI patients undergoing PCI. They observed focal FAPI uptake in all patients, with the affected myocardial FAPI uptake partially or completely matching the culprit lesion confirmed by coronary angiography. Furthermore, fibroblast activation volume (FAV) was strongly correlated with peak creatine kinase levels and negatively correlated with left ventricular function. Diekmann et al. (48) performed ^68^Ga-FAPI-46 PET imaging on 35 AMI patients within 11 days after revascularization. They found that FAPI uptake significantly exceeded the infarcted myocardium area and involved non-infarcted myocardium (Figure 2). Patients with higher FAPI uptake volume at baseline exhibited more severe left ventricular systolic dysfunction during follow-up. In addition, ^68^Ga-FAPI uptake did not completely correlate with myocardial tissue characteristics assessed by CMR, suggesting that the tissue fibrosis information provided by the two modalities might be complementary. A similar finding was reported by Xie et al. (49). In addition to exceeding the area of the infarcted area, FAPI uptake also exceeded the area of edema detected by MRI. Interestingly, in the region of coronary microvascular obstruction (MVO) identified by LGE, FAPI imaging demonstrated a low uptake area within a high uptake area, suggesting that FAPI imaging is not only able to detect infarcted regions but also identifies areas of microvascular obstruction, which is vital for further understanding of myocardial injury and repair processes. Following AMI, the typical feature of the fibrosis repair process is a significant increase in activated fibroblasts and myofibroblasts in the infarcted and border zones (14). Regardless of preclinical or clinical studies, the uptake extent of FAPI consistently exceeds the actual extent of the infarction, reflecting ongoing tissue repair. Some researchers suggest this indicates that FAP upregulation plays a role in reactive fibrosis, potentially affecting the non-infarcted myocardium (48). This interpretation is supported by an animal study (70). Alternatively, other researchers propose that FAP upregulation in the border zone may represent the migration of cardiac fibroblasts during inflammatory and proliferative phases, emphasizing the dynamic cellular activities involved in these processes (71). The precise relationship between the extent of FAPI and reactive fibrosis remains unclear and warrants further investigation.

To evaluate the value of FAPI PET in predicting late left ventricular remodeling in AMI patients, Zhang et al. (50) prospectively enrolled 26 AMI patients and performed FAPI PET imaging at baseline and 12 months later. Compared with baseline clinical characteristics, CMR imaging, and cardiac function parameters, FAPI uptake volume showed superior predictive ability in predicting late left ventricular remodeling 12 months after AMI (AUC = 0.938, *p* < 0.001). Moreover, different from preclinical studies, FAPI uptake in infarcted myocardium can still be detected 12 months post-MI, indicating the presence of activated myofibroblasts in the human heart involved in cardiac repair or LV remodeling for a much longer period than those in animal models.

FAPI PET has demonstrated significant potential in assessing early post-MI fibrosis activity and predicting post-MI cardiac remodeling, offering robust support for future clinical applications. Recent clinical studies have indicated FAPI uptake volume rather than FAPI uptake signal intensity as a prognostic indicator (48, 50). In the early stages following AMI, FAP signal intensity reflects the level of fibroblast activation, which may vary over time. In contrast, FAPI uptake volume represents the overall extent of myocardial fibroblast activation and is closely associated with the infarct size (50). Given that infarct size tends to remain relatively stable in the early phase of AMI, the FAPI uptake volume may exhibit less time-dependent variability. This characteristic may explain why FAPI uptake volume is a more reliable prognostic indicator for adverse left ventricular remodeling. However, despite these promising findings, there are still some challenges in the clinical application of FAPI PET imaging. Due to the substantial pathological resemblance between reparative and reactive fibrosis, distinguishing the two using FAPI PET imaging is challenging. Besides, owing to individual variations among MI patients, current research has not yet established a definitive FAPI uptake threshold for guiding timely intervention. Therefore, more research is needed in the future to clarify its clinical value in guiding MI patients.

#### 3.4.2 PET Imaging of αvβ3 integrin

αvβ3 integrin is a transmembrane cell surface receptor that promotes cell migration, proliferation, and interaction with the extracellular matrix, offering the potential for imaging angiogenesis and fibrogenesis. Expression of αvβ3 integrin is low in resting endothelial cells but is significantly upregulated in the state of intramyocardial angiogenesis after MI (58). In addition to endothelial cells, activated myocardial (myo)fibroblasts and macrophages also express αvβ3 during migration and chemotaxis (38, 39). αvβ3 molecular imaging is based on tracers containing an exposed RGD (arginine-glycine-aspartate) sequence peptides, which have a high affinity for binding to αvβ3 integrin. A range of PET tracers targeting αvβ3 integrin have been evaluated in experimental models of MI, including ^18^F-galacto-RGD (51–54), ^68^Ga-NODAGA-RGD (53, 55–57), ^18^F-AlF-NOTA-PRGD2 (64), ^68^Ga-TRAP(RGD)_3_ (53), ^68^Ga-DOTA-E[c(RGDfK)]_2_ (59–63) and ^18^F-Fluciclatide (58), some of which have been used in clinical applications (54, 57–59).

In preclinical animal studies, RGD-based PET tracers aggregated at the infarct and peri-infarct regions as early as 3 days, peaked at 1–3 weeks after coronary artery occlusion (51, 64), and high levels of tracer uptake in the infarcted zone early after MI were correlated with the absence of LV remodeling at 12 weeks (52). Jenkins et al. (58) demonstrated that ^18^F-fluciclatide uptake was increased in segments displaying functional recovery and associated with an increase in the probability of regional recovery in MI patients. In a recent study, ^68^Ga-NODAGA-RGD PET imaging was performed on 31 AMI patients within 3–14 days after PCI, and the results showed that the uptake of ^68^Ga-NODAGA-RGD in the ischemic and injured myocardium increased in the early stage after MI. Additionally, the uptake intensity of ^68^Ga-NODAGA-RGD was related to the improvement of global left ventricular function during the 6-month follow-up (57). Of note, Unlike FAPI imaging, high uptake of αvβ3-targeted tracers predicted recovery regions. This phenomenon may be related to the unique role of αvβ3 integrin in cardiac repair.

However, αvβ3 integrin upregulation occurs not only on activated fibroblasts but also on macrophages and micro vessel-activated endothelial cells. Therefore, its uptake may represent angiogenic, inflammatory, or fibrotic activity. Due to the difficulty in distinguishing the imaging signal from the cell type or specific pathological process, using αvβ3 integrin as a marker for fibrosis is constrained. In addition to αvβ3 integrin, activated fibroblasts can express multiple integrin subtypes (72). αvβ1 integrin is highly expressed on fibroblasts and plays a crucial role in the activation of latent TGF-β1, which is a pivotal factor in the pathogenesis of tissue fibrosis (73). Similarly, αvβ6 integrin is also highly expressed on activated fibroblasts, and blocking its expression reduces tissue fibrosis formation (74). Recently, ligands for other integrin subtypes have also been developed (75), which may be useful for targeted visualization of myocardial tissue fibrosis.

#### 3.4.3 PET imaging of collagen

Collagen (especially type I collagen) is a major component of the ECM, and targeting collagen synthesis itself is an excellent strategy to accurately and directly quantify post-MI fibrotic activity by observing the cumulative levels of active collagen. Molecular probes targeting collagen have important applications in the detection of fibrosis through various imaging techniques such as MRI and SPECT, and these probes have demonstrated their ability to image post-MI fibrosis (76). Accurate quantification is essential for assessing disease progression and response to therapy. Although MRI and SPECT have their respective strengths in specific areas, PET imaging is undoubtedly superior in accurately quantifying and dynamically monitoring collagen accumulation.

Kim et al. (65) developed two collagen-targeting tracers (^64^Cu-NOTA-Collagelin and ^64^Cu-NOTA-CRPA) and utilized them for dynamic PET imaging in MI rats. The findings of the study revealed that the washout kinetics of both ^64^Cu-labeled peptides and the non-specific control tracer ^64^Cu-DOTA in fibrous tissue were comparable, indicating a lack of specific binding of the ^64^Cu-labeled peptides to collagen. Hence, further enhancement of the affinity and specificity of collagen-binding ligands is required for effective collagen-targeted imaging *in vivo*. Collagen-targeted PET imaging for MI remains relatively scarce. However, there are relevant PET imaging applications for detecting fibrosis in other systems, providing techniques and experience for collagen-targeted PET imaging in post-MI settings.

Désogère et al. (77) conducted a study using ^68^Ga-CBP8, a tracer targeting type I collagen, in a mouse model of bleomycin-induced idiopathic pulmonary fibrosis (IPF). The study demonstrated higher tracer uptake in fibrotic lung regions compared to healthy controls. The researchers also analyzed ex vivo lung tissue samples from three IPF patients, observing a linear correlation between ^68^Ga-CBP8 signal and degree of fibrosis. Additionally, they found that ^68^Ga-CBP8 exhibited a greater affinity for newly formed collagen in active disease than mature, established collagen. This investigation validates the potential use of ^68^Ga-CBP8 for assessing active fibrosis. The peptide LRELHLNNN has been recognized as a selective binder to collagen I. Rosestedt et al. (78) designed two radiolabeled LRELHLNNN PET tracers, ^68^Ga-DOTA-PEG2-LRELHLNNN and^18^F-AlF-NOTA-PEG2-LRELHLNNN, and evaluated their distribution in healthy rats. Compared to ^18^F-AlF-NOTA-PEG2-LRELHLNNN, ^68^Ga-DOTA-PEG2-LRELHLNNN showed lower background binding in the liver. Therefore, they further evaluated this tracer in a mouse model of liver fibrosis. The results demonstrated that ^68^Ga-DOTA-PEG2-LRELHLNNN can specifically bind to hepatic fibrotic lesions *in vitro*, indicating its potential for PET imaging in detecting early-stage fibrosis. However, its low stability *in vivo* remains a challenge that needs to be addressed.

Collagen-targeted PET imaging has primarily been investigated in the context of liver and lung fibrosis, with relatively limited exploration in myocardial fibrosis. More preclinical or clinical studies are necessary to explore the applications of these probes in post-MI fibrosis.

#### 3.4.4 PET imaging of proline

Amino acid proline and its derivative hydroxyproline account for approximately one-quarter of the collagen composition (79). Proline is found almost exclusively in collagen, so it has great potential for clinical use in detecting active fibrosis. PET imaging of ^18^F-labelled proline derivatives ^18^F-fluoro-proline can recognize the process of collagen synthesis. However, it has four different stereoisomers: cis-L, cis-D, trans-L, and trans-D, which exhibit large differences in metabolism, uptake behavior, and biodistribution. D-isomers are less stable and have a lower affinity than the L-isomers, making the L-isomers a preferred probe for targeting collagen biosynthesis *in vivo* (80, 81). Balogh et al. (66) conducted a preclinical study of radionuclide-labeled proline in MI rats. PET imaging was performed on rats injected with trans- or cis-4-18F-fluoro-L-proline one week after infarction. The results showed that uptake of trans-proline was accumulated in the infarcted area. Trans-proline SUVr was strongly inversely correlated with ventricular ejection fraction (r2 = 0.93), scar tissue size as measured by magnetic resonance imaging, and fibrotic regions as determined by PSR staining. To date, apart from this study, no other relevant studies have been conducted on PET imaging of proline in MI. More research and experimental data are still needed to fill this shortage in the future.

#### 3.4.5 PET imaging of other fibrosis-related targets

Several regulatory systems are involved in post-MI fibrosis, including the activation of the renin-angiotensin system, thrombin-activated Factor XIII (FXIII), and the activation of matrix metalloproteinases (MMPs).

In the renin-angiotensin system, AngⅡ is one of the main active substances. It can bind to AT1R on myofibroblasts and promote their proliferation, migration, and induction of ECM synthesis and deposition (82). AT1R has been considered as a possible target for imaging myofibroblasts. In a study by Fukushima et al. (67), probe ^11^C-KR31173 targeted at angiotensin receptors was evaluated in a pig model of MI. After MI, tracer myocardial retention increased in infarcted and non-infarcted regions compared with healthy controls. Similarly, the study also conducted initial clinical imaging trials to demonstrate the feasibility and safety of imaging. While the tracer uptake in the human body was lower than the level observed in the pig experiments, the retention of the tracer was sufficient for PET imaging.

Thrombin­activated FXIII also plays an important role in post-MI fibrosis, mediating the deposition and cross-linking of collagen after tissue damage (83). The feasibility of using ^111^In-DOTA-FXIII for targeted SPECT imaging of FXIII has been validated in a mouse model of MI (83). In this experiment, ^111^In-DOTA-FXIII was cross-linked by FXIII to extracellular matrix proteins and accumulated in regions of increased FXIII activity. While SPECT imaging has demonstrated the feasibility of targeting factor FXIII, there remains a significant gap in the development of PET probes. Moreover, in recent years, there has been a lack of research focused on using FXIII activity detection to evaluate myocardial fibrosis, and its potential value in this context still requires further investigation and validation.

Matrix metalloproteinases (MMPs) are a family of zinc-dependent proteinases involved in cardiac fibrosis and scar formation (84). When collagen deposition increases in cardiac tissue, MMPs exhibit higher activity levels, promoting excessive collagen hydrolysis and degradation. This increased activity of MMPs suggests that they may serve as biomarkers for early cardiac fibrosis formation (85). The SPECT tracer ^99m^Tc-RP805, which specifically binds to MMPs, was imaged *in vivo* in a porcine model of MI (86). The retention of the tracer in the infarct region peaked with a fourfold increase one week post-MI and remained elevated for up to four weeks. Radiotracer uptake was correlated with MMPs activity and predicted late post-MI LV remodeling. A novel PET radioisotope-labeled probe, ^64^Cu-RYM2, has been designed to target MMPs and has been evaluated in a murine model of abdominal aortic aneurysm (AAA) as well as in human aortic tissue (87). The study demonstrated that ^64^Cu-RYM2 exhibits good stability, rapid renal clearance, and specific binding to MMPs, which closely correlated with MMP activity. Considering the crucial role of MMPs in the fibrotic process following MI, ^64^Cu-RYM2 may also have potential value in the clinical setting of MI.

## 4 Summary and future directions

Myocardial fibrosis is part of the repair process of the myocardium after MI. However, this repair process may become dysregulated in some cases, leading to excessive fibrosis and adverse cardiac remodeling. Despite the clear association between myocardial fibrosis and poor prognosis in MI patients, developing antifibrotic strategies remains challenging due to the heterogeneity and complexity of its pathophysiology. Therefore, it is vital to screen suitable individuals and administer antifibrotic therapy at the optimal time. PET imaging of different targets can reflect various stages of early post-MI myocardial fibrosis, which cannot be achieved with conventional imaging methods. Molecular probes represented by FAPI can assess the extent and distribution of early fibrosis accurately and in real time, providing valuable prognostic information. In contrast to targeted FAP and integrin PET imaging, other molecular imaging techniques lack empirical support for clinical applications. Notably, ECM remodeling is a relatively late pathological process in myocardial fibrosis after MI. Therefore, PET imaging of collagen and its precursor proline, a late product of fibroblast activation, may offer limited clinical benefit for patients. In the future, more clinical studies are required to explore the exact clinical value of fibrotic PET imaging for guiding MI. Further research should focus on exploring more targets and developing the most promising imaging tracers, which can bring breakthroughs in diagnostic accuracy and the development of individualized therapies, ultimately improving the prognosis of patients.
